# Sudapyridine (WX-081) inhibits *Mycobacterium tuberculosis* by targeting ATP synthase and upregulating host innate immunity

**DOI:** 10.1128/msphere.00149-25

**Published:** 2025-05-21

**Authors:** Xinda Li, Xiaoyi Luo, Bin Wang, Lei Fu, Xi Chen, Yu Lu

**Affiliations:** 1 Department of Pharmacology, Beijing Chest Hospital, Capital Medical University 12517, Beijing, China; 2 Beijing Key Laboratory of Drug Resistance Tuberculosis Research, Beijing Tuberculosis and Thoracic Tumor Research Institute 117550 , Beijing, China; Washington University in St. Louis School of Medicine, St. Louis, Missouri, USA

**Keywords:** WX-081, drug-resistant tuberculosis, ATP synthase, immunomodulation

## Abstract

Drug-resistant tuberculosis (DR-TB) urgently requires safer, more accessible alternatives to bedaquiline (BDQ), which faces critical flaws like cardiotoxicity, high costs, and emerging resistance. WX-081, a promising BDQ alternative, has demonstrated superior anti-TB activity and improved safety in clinical studies. However, its mechanism of action remains unexplored, underscoring the need for further research to optimize its potential in advancing global TB elimination efforts. This study reveals WX-081’s dual mechanisms: targeting *atpE* to disrupt ATP synthase and proton motive force via resistance screening, gene sequencing, and functional assays while enhancing host immunity through macrophage transcriptomics. Molecular docking confirmed *atpE* binding sites, and immune activation pathways (NF-κB/MAPK) were identified, positioning WX-081 as a potent, safe anti-DR-TB candidate despite unresolved mechanistic details.

**IMPORTANCE:**

Bedaquiline, a key drug for drug-resistant tuberculosis, is restricted by safety issues impacting its clinical utility. Its next-generation alternative, WX-081, has advanced to Phase III trials but lacks in-depth studies on its mechanism and host immune-modulatory effects, necessitating further research before broad clinical adoption.

## INTRODUCTION

Tuberculosis (TB), a formidable infectious disease caused by *Mycobacterium tuberculosis* (Mtb), has long been the leading cause of death among infectious pathogens, which imposes a heavy burden on public health worldwide (1–3). The World Health Organization (WHO) has proposed a strategy to end TB by 2035, which aims to reduce the TB incidence by 90% and TB-related deaths by 95% in 2035 compared to 2015 levels (4–6). However, the Global Tuberculosis Report 2024 highlighted that an estimated 10.8 million people worldwide contracted TB in 2023, with only an 8.3% reduction in TB incidence between 2015 and 2023. The global number of TB-related deaths in 2023 was about 1.25 million (4, 7, 8). In short, we still have a long way to go to end TB completely.

Drug-resistant tuberculosis (DR-TB) is caused by the mutation of Mtb that leads to resistance to one or more of the standard anti-TB drugs (9, 10). Treating DR-TB requires the use of second-line drugs, which are less effective, more toxic, and costlier than first-line medications. Additionally, the treatment duration can extend to 18–24 months, compared to the 6-month regimen for drug-susceptible TB (11–14). The continuous emergence of DR-TB has gradually become a key factor restricting the end of the TB epidemic. For decades after the approval of rifampin in 1962, no new anti-tuberculosis drugs were successfully developed, until bedaquiline (BDQ) was approved by the FDA for the treatment of drug-resistant tuberculosis in 2012 (15). BDQ, a diarylquinoline antibiotic, targets the proton pump of the ATP-synthase enzyme in mycobacteria, resulting in severe impairment of energy synthesis and death (16–19). In 2018, BDQ was listed by WHO as the first recommended drug for the treatment of rifampicin-resistant TB and multidrug-resistant TB (MDR-TB). However, the use of BDQ has been severely limited by its toxicity, including unexplained deaths, QTc prolongation, phospholipidosis, and hepatotoxicity (16, 20–24). Therefore, there is an urgent need for safer and more potent drugs related to BDQ.

Sudapyridine (WX-081) is a new and excellent diarylquinoline analog, which is currently in Phase III clinical trial (CTR20221162) (25). Compared with BDQ, WX-081 has improved safety, higher pulmonary exposure, and lower risk of QT interval prolongation, while it still retains strong activity similar to bedaquiline against both drug-susceptible and drug-resistant Mtb (26). Besides, WX-081 also showed remarkable activity against non-tuberculous mycobacteria (27). WX-081 is a potential alternative to bedaquiline in the treatment of drug-resistant tuberculosis, but its targeting mechanism on Mtb and its immunomodulatory effects on the host have not been studied.

In this study, we identified that WX-081 inhibits mycobacterial ATP synthesis by targeting ATP synthase and disruption of proton motive force (PMF). WX-081 also activates innate immunity by inducing cytokines and type I interferons in response to infection with WX-081-resistant strains and lipopolysaccharide (LPS) stimulation. Overall, our work provides detailed insights into the mechanisms of action of WX-081, a promising candidate for the treatment of drug-resistant tuberculosis.

## RESULTS

### Mutations of *atpE* occur in WX-081 stepwise induction resistant isolates

Mutations in the gene encoding the putative cellular target of the drug confer resistance, thus helping the investigation of the mechanism of action of antimicrobials. To elucidate the putative mechanism of action of WX-081, we performed stepwise induction of WX-081-resistant strains by inoculating H37Rv in logarithmic growth phase onto 7H10 medium containing 1× MIC of WX-081, followed by transferring single colonies onto plates containing twofold serially increasing concentrations of WX-081, up to 16× MIC. Finally, single colonies were collected for Sanger sequencing. In previous studies, BDQ-resistant strains mainly had mutations in three genes, *atpE*, *Rv0678*, and *pepQ*. The mutation of *atpE* prevents BDQ from binding to the c subunit of ATP synthase, thus impairing the function of the ATP synthase complex (28, 29). *Rv0678* and *pepQ* are both associated with the efflux pump mmpS5-mmpL5, and mutations in *Rv0678* and *pepQ* lead to enhanced drug efflux, but are not specific to BDQ and are also less sensitive to other drugs, indicating that they are not direct targets of BDQ (30–32).

A total of 10 resistant strains were obtained by this method, and the *atpE*, *Rv0678,* and *pepQ* of these strains were sequenced. We found that all isolates exhibited dual mutations in *atpE* and *Rv0678*. Specifically, four isolates carried Ala63Val, three isolates had Ala63Thr, and three isolates showed Ala63Pro mutations in *atpE* (Table 1). *pepQ* was not mutated in any of these 10 strains. The MICs of these resistant strains to BDQ were also examined and showed an 8–32-fold increase.

**TABLE 1:** MIC determination and genotypic characterization of drug-resistant induction strains

Gene	Missense mutation (DNA/amino acid) in *atpE*	Missense mutation (DNA/amino acid) in *Rv0678*	No. of isolates	MIC (μg/mL)	
				WX-081	BDQ
*atpE + Rv0678*	C188T/A63V	G71A/G24D	3	2	0.5
	G187A/A63T	G197A/G66E	1	2	0.5
	G187A/A63T	G196A/G66E	2	2	0.5
	G187C/A63P	G74A/G25D	2	2	2
	G187C/A63P	G196A/G66E	1	2	0.5
	C188T/A63V	G196A/G66E	1	2	0.5

### Spontaneous resistant isolates to WX-081 also harbor the mutations in *atpE*

In addition, spontaneous resistance mutants to WX-081 were obtained by directly exposing Mtb to 4× or 8× MIC of WX-081. In total, we obtained 20 resistant strains, which were also sequenced. Sequencing information showed that a total of 17 strains had mutations in *RV0678*, of which three strains had mutations in the *atpE* gene, and the other three strains had no mutations in the above three genes (Table 2). We also determined the MICs of these resistant strains to BDQ, which showed a decrease in sensitivity and an increase in MIC to 8–16 times that of the wild-type susceptible H37Rv strain.

**TABLE 2:** Genotypic characterization of spontaneous resistance isolates to WX-081

Gene	Missense mutation (DNA/amino acid) in *atpE*	Missense mutation (DNA/amino acid) in *Rv0678^b^*	No. of isolates
*Rv0678*		C466T/R155^*a*^	4
		T80del	1
		C467 ins	2
		GGA165 ins	1
		G74A/G25D	1
		C296T/A99V	1
		G417T/M139I	1
		G281T/R94L	1
		G198 del	1
		G398 del	1
*Rv0678 + atpE*	G187C/A63P	A444C/N148H	1
	G187C/A63P	G84T/E28^*a*^	1
	G187C/A63P	G417T/M139I	1
None	Wild type	Wild type	3

^*a*^ Stop codon.

^*b*^ del, deletion; ins, insertion.

To verify the cross-resistance between WX-081 and BDQ, we detected the MICs of WX-081 against BDQ-resistant strains using the microplate alamar blue assay (MABA) method. As shown in Table 3, the MIC values for WX-081 against the two BDQ-resistant strains with specific *atpE* missense mutations were 2 µg/mL, which was 16-fold higher than MIC against susceptible strains, indicating cross-resistance between BDQ and WX-081. BDQ also had an MIC of 2 µg/mL against all these resistant strains, suggesting that WX-081 also shares similar mechanisms of action with BDQ. Taken together, these data indicate that the target of WX-081 most likely remains the *atpE* gene of ATP synthase.

**TABLE 3:** Determination of cross-resistance between WX-081 and BDQ^*a*^

Strain	Mutation		MIC (μg/mL)			
	*Rv0678*	*atpE*	WX-081	BDQ	CFZ	RFP
H37Rv			0.125	0.06	0.25	0.03
BDQ 1000-0.125B	A193 ins	A83G	2	2	0.125	0.015
BDQ 1000-1B	G193 ins	A83G	2	2	0.125	0.015

^*a*^ ins, insertion; CFZ, clofazimine; RFP, rifampicin.

### WX-081 interferes with mycobacterial energy metabolism by blocking *atpE* synthase

**Fig 1:**
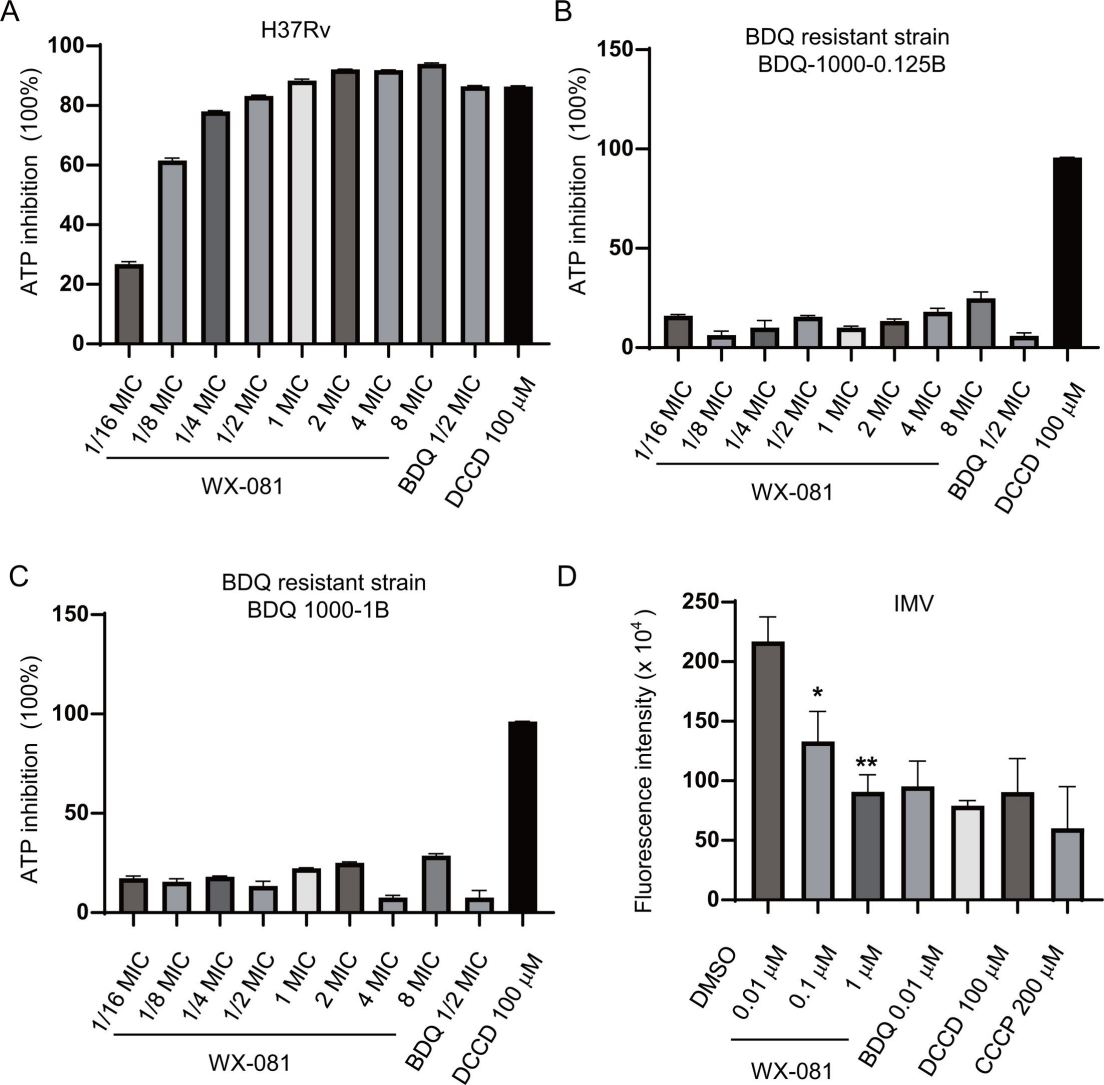
WX-081 inhibits ATP synthesis via inhibiting ATP synthase and destroying proton motive force. (A–C) Intracellular ATP levels in Mtb after 24 h compound treatment: drug-susceptible H37Rv (**A**), BDQ-resistant strains BDQ 1000-0.125B (**B**), and BDQ 1000-1B (**C**). (**D**) ATP synthesis inhibition in *Mycobacterium smegmatis* inverted membrane vesicles (IMVs). (*n* = 3, means and standard deviation [SD], *P < 0.05, two-sided *t*-test).

Given the above findings, WX-081 may block *atpE* as its main target. To further clarify the mechanism of action of WX-081, we first examined the effect of WX-081 on ATP production. To this end, Mtb was treated with different concentrations of WX-081 for 24 h, and the cellular ATP levels were detected. The results showed that WX-081 with concentrations from 1/16× MIC to 8× MIC, as well as BDQ, all could dramatically inhibit the synthesis of ATP of H37Rv and showed a dose-dependent decrease (Fig. 1A). ATP synthase activity was completely blocked by the specific inhibitor N,N′-dicyclohexylcarbodiimide (DCCD), as a positive control. In contrast, no significant inhibition was observed in BDQ-resistant strains (*atpE*^D28G^) treated with WX-081 (Fig. 1B and C).

In addition, the inverted membrane vesicles (IMVs) were generated from *Mycobacterium smegmatis* mc^2^ 155 (ATCC 700084) as described previously (33). We examined the ATP synthesis under WX-081 treatment. In accordance with the changes observed in the cells, WX-081 (0.01–1 μM) and BDQ inhibited ATP levels in the inverted vesicles compared to the dimethyl sulfoxide (DMSO) group. (Fig. 1D). Altogether, these results suggested that WX-081 interferes with mycobacterial energy metabolism, leading to the decrease of ATP abundance.

### WX-081 displays protonophore activity to collapse the transmembrane pH gradient

**Fig 2:**
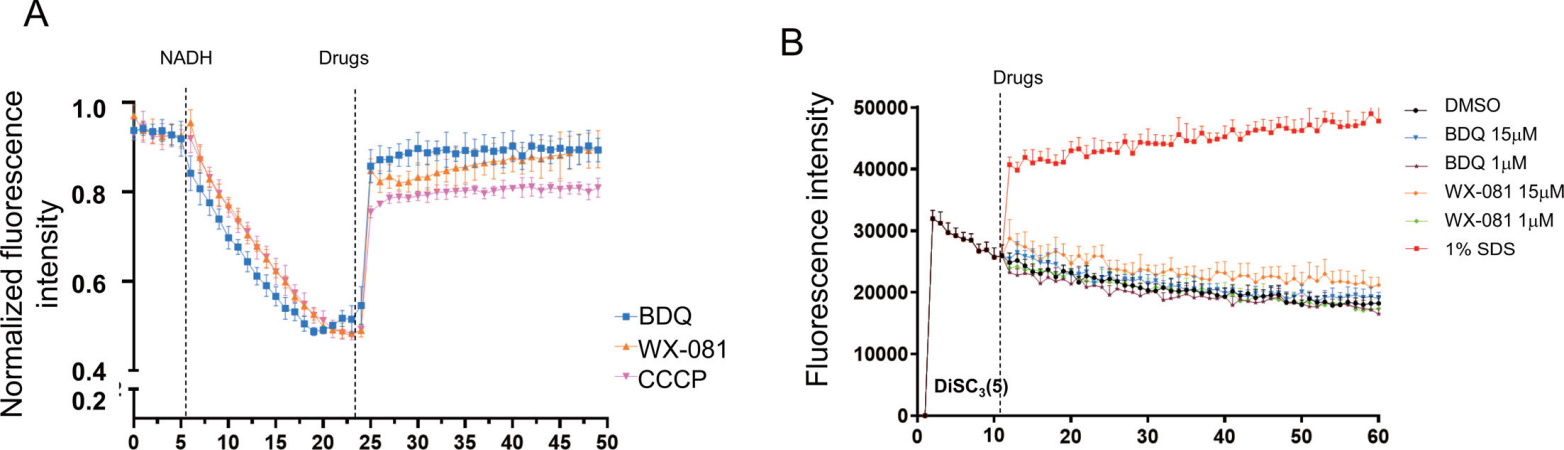
WX-081 displays protonophore activity to collapse the transmembrane pH gradient. (A and B). WX-081’s impact on transmembrane proton gradient (ΔpH) and membrane potential (Δψ) in IMVs, assessed using pH-sensitive fluorescent dye ACMA (**A**) and membrane potential probe DiSC_3_(5) (**B**), respectively. Positive controls: CCCP (ΔpH collapse) and 1% SDS (Δψ disruption). (*n* = 3, means and SD, *P < 0.05, two-sided *t*-test).

Recently, BDQ was uncovered to be an H+/K+ antiporter (34, 35). Through its protonophore activity, the drug shuttles protons across the lipid bilayer to collapse the transmembrane pH gradient component of the proton motive force. Elimination of the pH gradient disables the link between electron transport and ATP synthesis (34–36). BDQ’s uncoupler activity appears to be a second mechanism of action of the drug, contributing to the drug’s bactericidal activity against Mtb (35, 36). To determine whether WX-081 had protonophore activity, we measured the effect of WX-081 on the transmembrane pH gradient of *Mycobacterium smegmatis* inverted vesicles using the pH-responsive fluorophore 9-amino-6-chloro-2-methoxyacridine (ACMA) as described previously (36). NADH was used as an electron donor to energize the vesicles. The addition of 5 mM NADH resulted in quenching of ACMA fluorescence, thus indicating the establishment of the transmembrane pH gradient. WX-081 caused a dose-dependent reduction of ACMA fluorescence quenching. BDQ and uncoupler CCCP were used as positive controls (Fig. 2A).

PMF is collectively established by two parameters: pH gradient (ΔpH) and membrane potential (Δψ). To investigate the effect of WX-081 on membrane potential (Δψ), the membrane potential-sensitive dye DiSC_3_(5) was used. WX-081 did not lead to significant changes in Δψ (Fig. 2B), and a similar result was observed with BDQ, which was consistent with previous studies on BDQ and analogs (37). SDS (1%) was used as a positive control and rapidly depolarized the membrane potential of Mtb. Altogether, these results suggested that WX-081 collapses the components of the mycobacterial PMF by eliminating the pH gradient to uncouple electron transport from ATP synthesis.

### Molecular docking reveals the binding site between WX-081 and *atpE*

**Fig 3:**
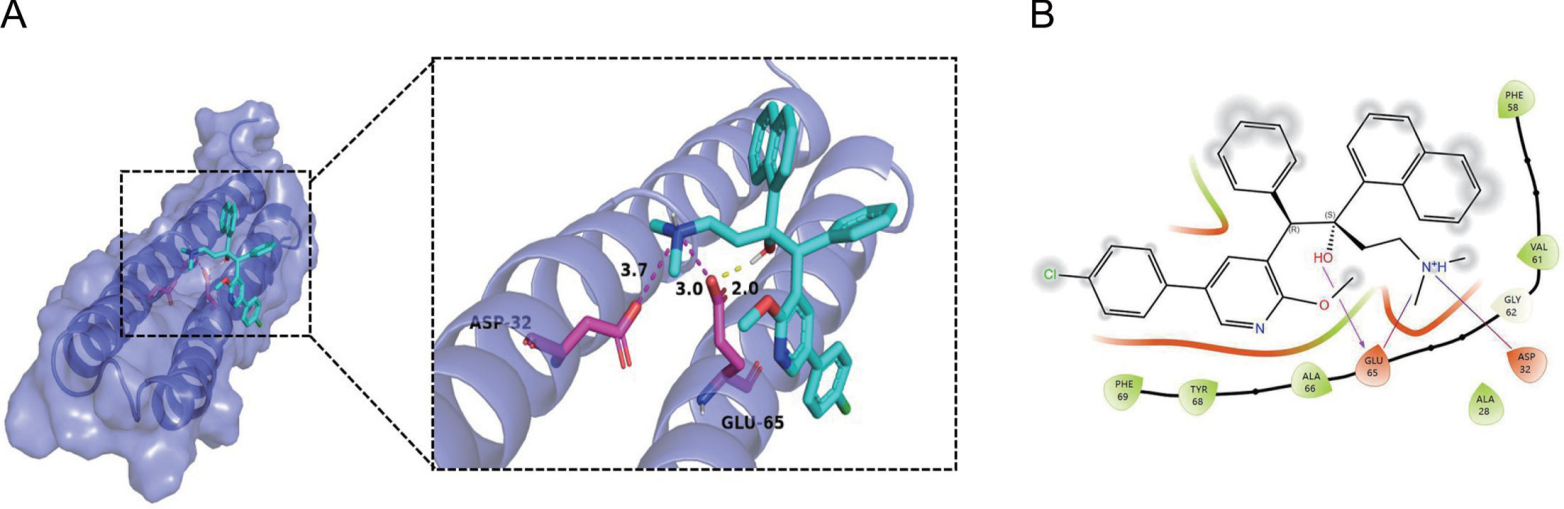
Interactions between Mtb ATP synthase *atpE* and WX-081. (**A**) Molecular interactions between WX-081 and *atpE* (ATP synthase subunit c) at predicted binding sites. (**B**) Two-dimensional interaction map of WX-081 with *atpE*. The *atpE* protein is depicted as a slate-colored cartoon model, while WX-081 (cyan stick) binds to critical residues (magenta sticks) within the subunit. Nonpolar hydrogen atoms are omitted for clarity. Hydrogen bonds and ionic interactions are highlighted by yellow and magenta dashed lines, respectively.

Genetic analysis of the WX-081 mutant-resistant strains suggests that mutations in the *atpE* gene, c subunit of ATP synthase, are its main target, similar to BDQ. We also used the docking technique to investigate the potential binding sites between WX-081 and *atpE* (PDB: 4V1F). In the modeling of WX-081-bound *atpE*, the interactions that anchor WX-081 are involved with the residues Asp32 and Glu65. Hydrogen bond interactions were observed between *atpE* Glu65 and WX-081. In addition, residues Asp32 and Glu65 of th*e atpE* protein are involved in ionic interactions with WX-081 (Fig. 3A and B). These results are similar to those observed in modeling of BDQ and analog drugs (38, 39). Therefore, it also indicated that WX-081 targets *atpE*.

### WX-081 promotes innate immune signaling pathways

**Fig 4:**
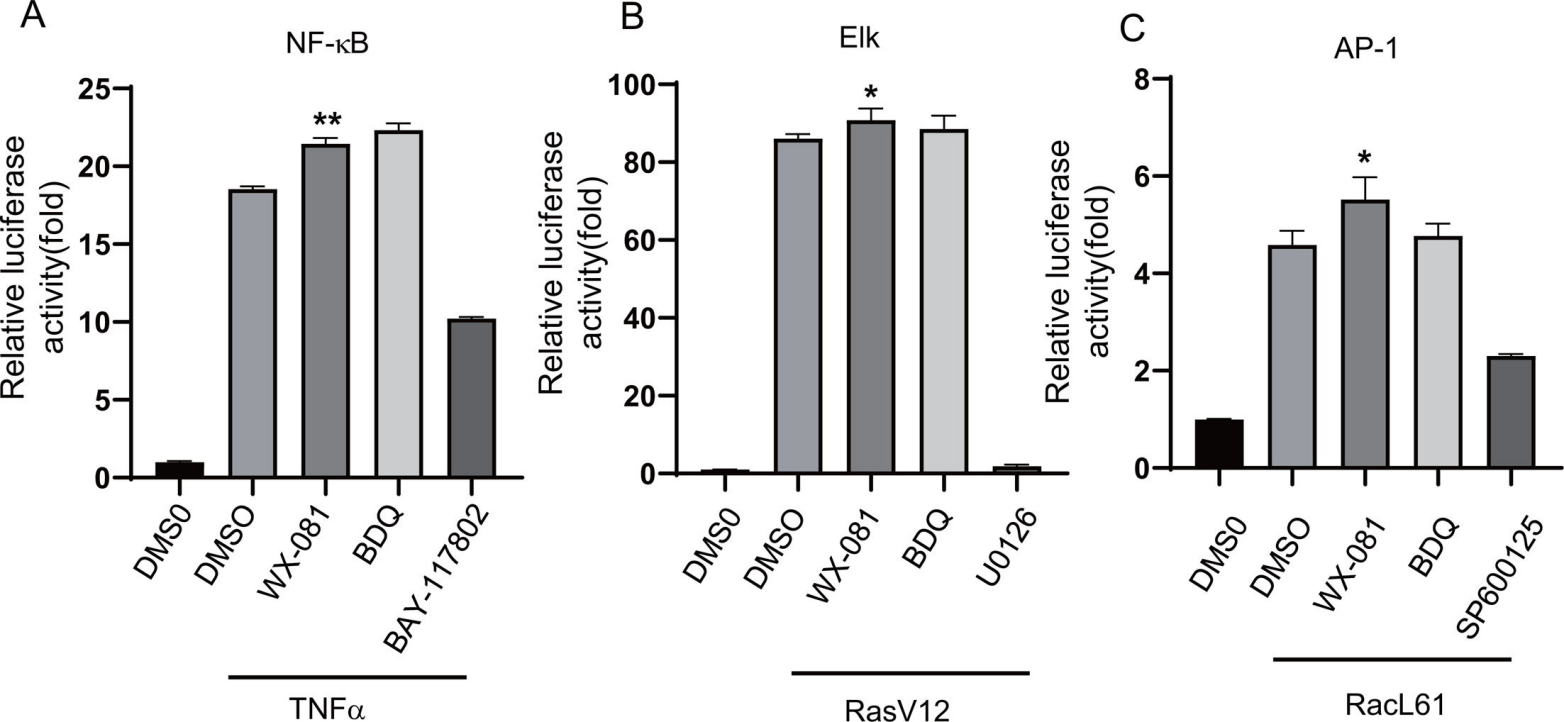
WX-081 promotes the activation of innate immune signaling pathways. (A–C) Luciferase assays of HEK293T cells transfected with plasmids encoding NF-κB (A), JNK (B), or ERK (C) luciferase reporters, and treated with WX-081. The ERK pathway was activated by co-expression of constitutively active RasV12. The JNK and p38 MAPK pathways were activated by co-expression of constitutively active RacL61. The NF-κB pathway was stimulated by TNF treatment. BAY-117082, U0126, and SP600125 were used as positive controls. (*n* = 3, means and SD, *P < 0.05, **P < 0.01, two-sided *t*-test).

The host immune system plays an important role in the clearance of Mtb and tissue damage repair. In recent years, more and more studies have found that antibiotics used in the treatment of tuberculosis not only have their own antibacterial effects but also have different regulatory effects on the immunity of the host, which may affect their efficacy. To assess whether WX-081 influences host innate immunity, we utilized the NF-κB and MAPK dual luciferase reporter system for an initial evaluation. HEK293T cells were transfected with NF-κB and MAPK dual luciferase reporter plasmids and treated with WX-081. The cells were harvested for the assay of luciferase activity. We found that WX-081 significantly activated both NF-κB and MAPK signaling pathways. In addition, we also found for the first time that BDQ also exerted a strong activation effect on innate immunity (Fig. 4A through C).

### WX-081 increases the expression of cytokines and type I interferon

**Fig 5:**
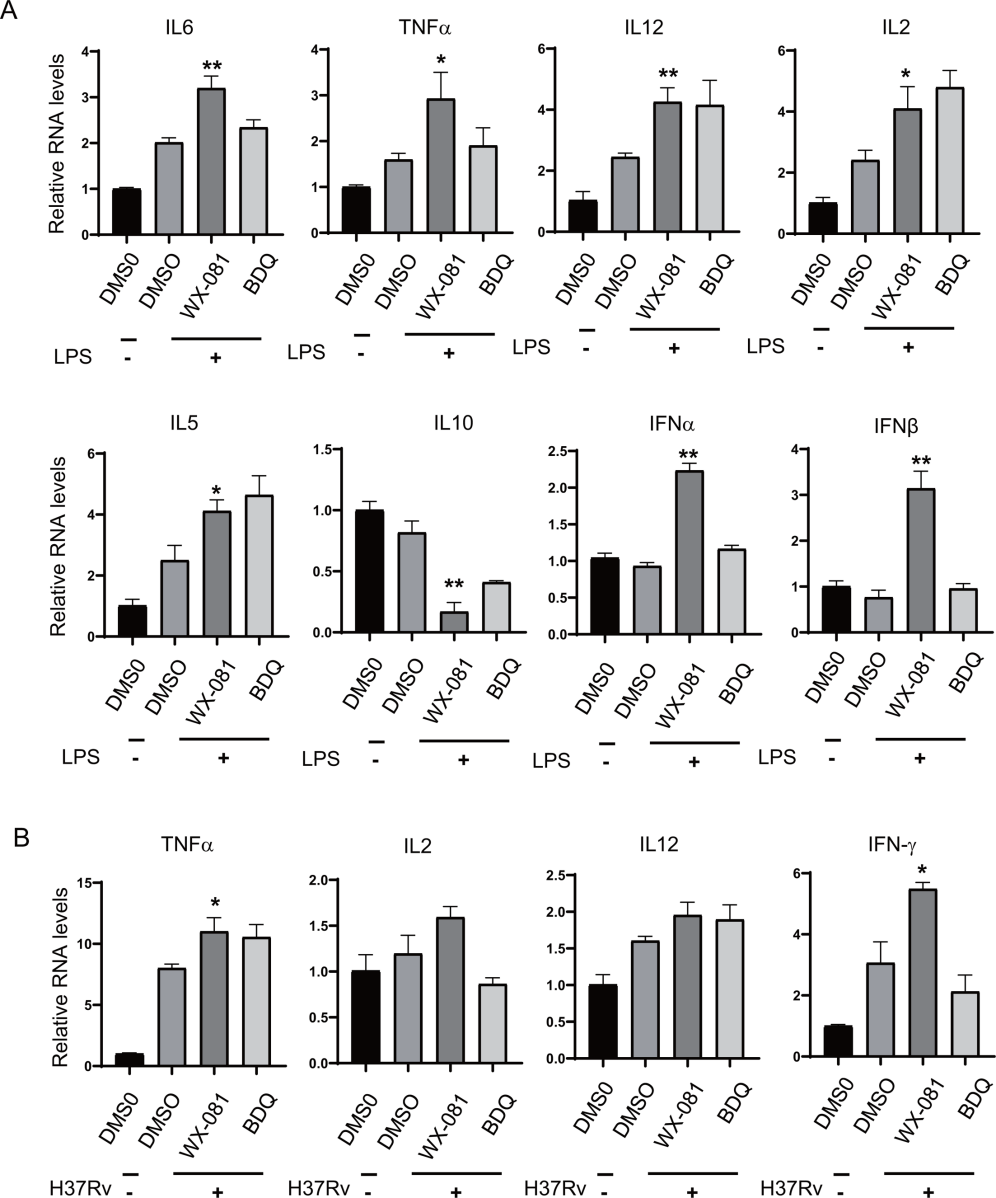
WX-081 improves the expression of cytokines and type I interferon. (**A**) Quantitative PCR analysis of cytokines and type I interferon mRNA expression in J774A.1 macrophages pretreated with WX-081 or BDQ for 12 h and stimulated with LPS (100 ng/mL) for another 12 h. (**B**) RNA levels in J774A.1 macrophages infected with H37Rv (multiplicity of infection [MOI] = 5) for 4 h and treated with WX-081 or DMSO for another 24 h. (*n* = 3, means and SD, *P < 0.05, two-sided *t*-test).

Macrophages are the main survival site of the host after Mtb infection. As an important member of the innate immune system, macrophages are also the main source of cytokines and play an important role in the activation of other immune cells and adaptive immunity. To identify the effect of WX-081 on cytokine production, J774A.1 macrophages were stimulated with LPS and incubated with WX-081 or BDQ for 24 h, first. The expression of inflammatory cytokines and type I interferon was analyzed through Real-time quantitative polymerase chain reaction (RT-qPCR). Under LPS stimulation, the results showed that WX-081 significantly promoted the levels of interleukin-6 (IL-6), interleukin-12 (IL-12), interleukin-2 (IL-2), interleukin-5 (IL-5), and tumor necrosis factor-α (TNF-α) mRNA expression relative to DMSO control, as well as IFNα and IFNβ, while decreasing the level of IL-10, a key immunosuppressive factor (Fig. 5A).

**Fig 6:**
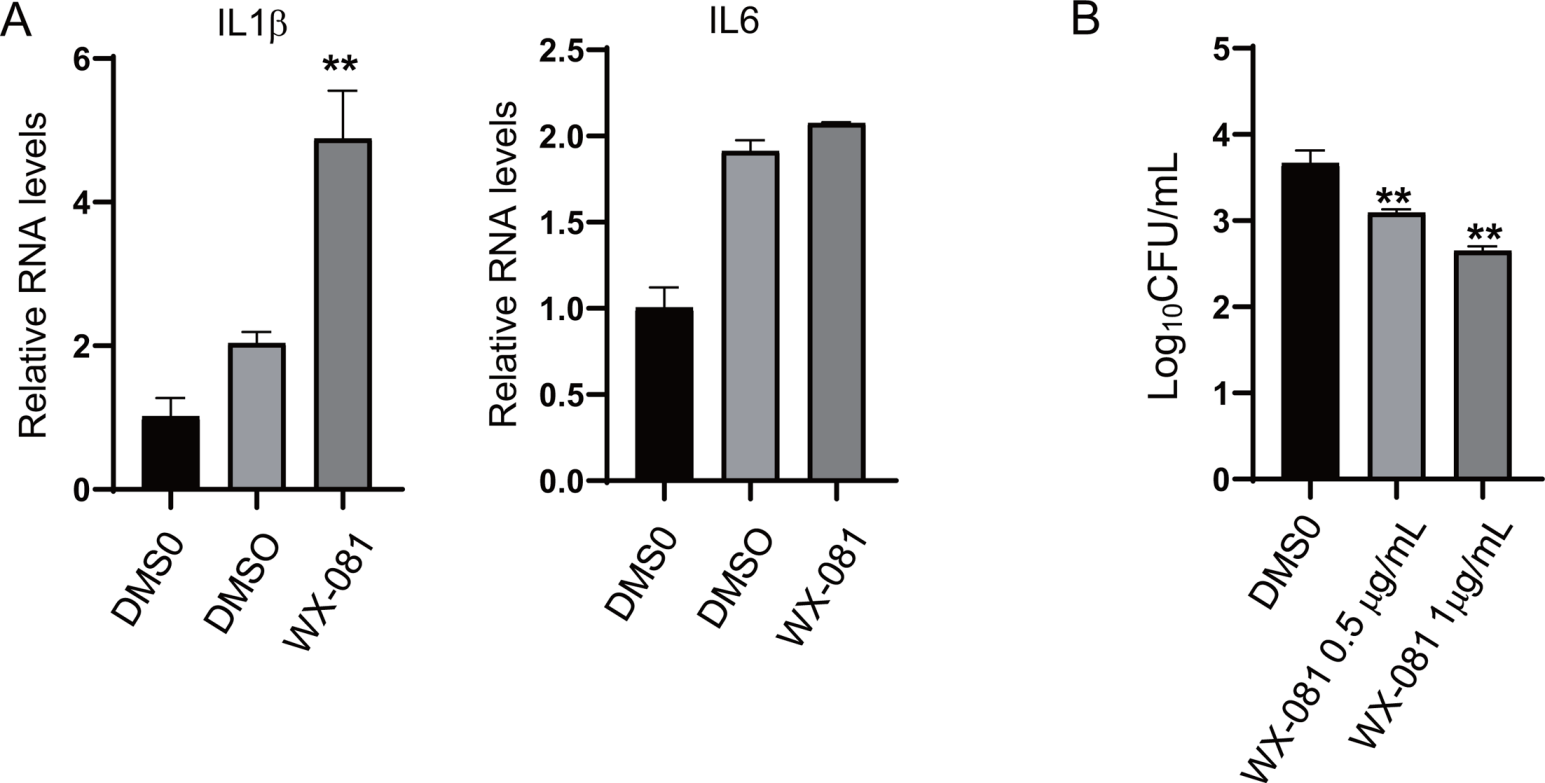
WX-081 inhibits the intracellular growth of WX-081r-Mtb. (**A**) RNA levels in J774A.1 macrophages infected with WX-081r-Mtb (MOI = 5) for 4 h and treated with WX-081 or DMSO for another 24 h. (**B**) Intracellular colony-forming units (CFUs) assay in mouse macrophage J774A.1 infected with WX-081r-Mtb (MOI = 5) for 4 h and treated with WX-081 or BDQ for another 48 h. (*n* = 3, means and SD, *P < 0.05, two-sided *t*-test).

In addition, J774A.1 macrophages were infected with H37Rv (multiplicity of infection [MOI] = 5), and the mRNA expression of cytokines TNF-α, IL-2, IL-12, and IFNγ was detected, which was consistent with the results of LPS treatment. WX-081 promoted the expression of TNF-α, IL-2, IL-12, and IFNγ in comparison to the DMSO control (Fig. 5B). We also infected cells with a resistant strain of WX-081 (WX-081r-Mtb), and WX-081 treatment also led to the upregulation of the cytokines IL-6 and IL-1β (Fig. 6A). In conclusion, WX-081 enhances host innate immunity during Mtb infection, causing upregulation of cytokines and type I interferon expression.

### The immune activation of WX-081 enhances its intracellular bactericidal activity

To determine whether innate immunity was involved in this antibacterial activity, we thus asked whether WX-081 conferred protection against bacterial infections naturally resistant to WX-081. J774A.1 cells were infected with WX-081r-Mtb for 4 h and then incubated with WX-081 for 48 h. We quantified bacterial colony-forming units (CFUs) in the different treatment groups and found that the bacterial load in cells infected with WX-081r-Mtb was significantly reduced when treated with WX-081 (Fig. 6B). Therefore, these data revealed that WX-081 activates macrophage bactericidal functions through upregulating antibacterial immunity.

### RNA sequencing indicates that WX-081 promotes innate immunity

**Fig 7:**
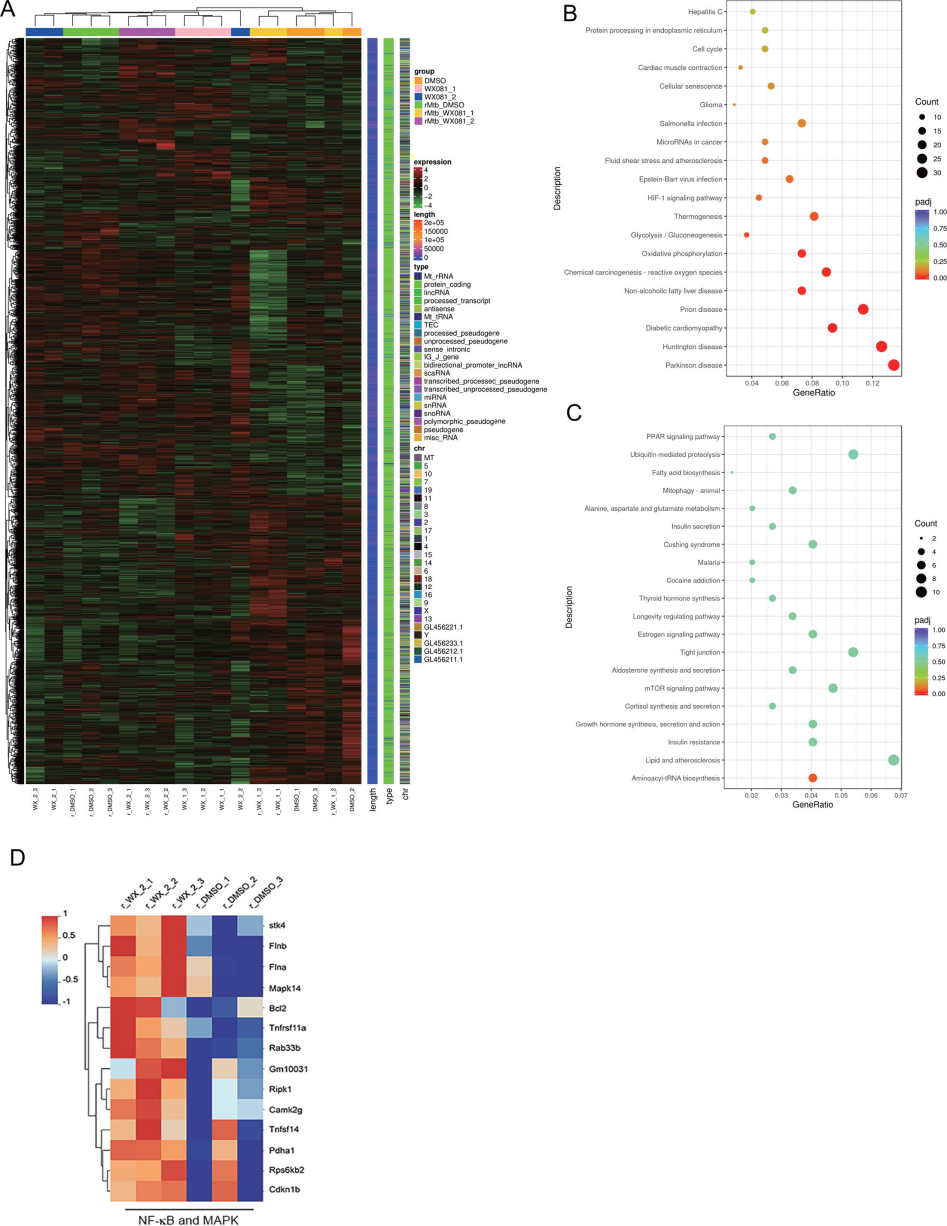
RNA deep sequencing reveals that WX-081 upregulates host innate immunity against Mtb. (**A**) The DEGs in J774A.1 cells infected with WX-081r-Mtb (MOI = 5) and treated with WX-081 (0.5 µg/mL), WX-081 (1 mg/mL), or DMSO were analyzed by RNA-seq. The heat map shows the differentially expressed genes in all groups (B and C) The KEGG analysis of WX-081 compared with DMSO groups. Shown are the signal pathways enriched by upregulated genes (B) or downregulated genes. (**D**) Heat maps showing transcripts with differential expression in macrophages treated with WX-081 or DMSO under infection with WX-081r-Mtb, associated with NF-κB/MAPK signaling pathways.

To further investigate the effect of WX-081 on the activation of innate immunity in J774A.1 macrophages, RNA deep sequencing was performed. Macrophages were infected with WX-081r-Mtb at an MOI of 5 for 4 h. Subsequently, either 0.5 µg/mL or 1 µg/mL WX-081 was added to the culture, followed by another 24 h of incubation. Differentially expressed genes (DEGs) with false discovery rate (FDR) corrected *P* value (padj) ≤ 0.05 were identified among all group comparisons. The differential genes of all treatment groups are presented in the heatmap (Fig. 7A). We performed Gene Ontology (GO), Kyoto Encyclopedia of Genes and Genomes (KEGG), and Reactome enrichment analysis for DEGs. According to KEGG analysis, WX-081 upregulated genes were enriched in many innate immune pathways, including the HIF-1 signaling pathway, reactive oxygen species (ROS) production, and glycolysis/gluconeogenesis (Fig. 7B). mTOR was enriched in the downregulated signaling pathways (Fig. 7C). Intracellular ROS are important intracellular bactericidal mediators. HIF1 plays an important role in regulating the production of cytokines and IFNγ. It also mediates the immunometabolic reprogramming and induces the increase of glycolysis during Mtb infection. In the WX-081 treatment group, genes associated with the NF-κB and MAPK innate immune signaling pathways exhibited significant upregulation, including Stk4, Mapk14, Tnfrsf11a, RIPK1, and Tnfsf14.

In the infected WX-081r-Mtb group, the expression of genes such as Mapk14 was significantly increased in the WX-081 group compared to the DMSO group, and it has been shown that the activation of MAPK was associated with the activation and differentiation of Th1 cells, leading to the production of intrinsic immune system cytokines such as TNF, IL-6, IL-12, IL-1β essential for defense against *Mycobacterium tuberculosis*, which is consistent with our results (40). Compared to the DMSO group, the WX-081-treated group significantly upregulated the Tnfrsf11a gene which encodes a protein that is a member of the TNF receptor superfamily, through which activation of NF-κB and MAPK/JNK is induced (Fig. 7D). TNF cytokine is one of the cytokines that are closely associated with Mtb and are essential for controlling infection (41).

In summary, we have identified two mechanisms of action for WX-081. First, it targets *atpE*, disrupting the PMF and thereby affecting the operation of ATP synthase. Second, it indirectly upregulates the host’s innate immune response, enhancing intracellular bactericidal activity.

## DISCUSSION

DR-TB poses a significant challenge to global health due to its complexity and difficulty in treatment (42, 43). Current therapies, including the use of BDQ, a relatively recent addition to the DR-TB arsenal, are fraught with limitations. While BDQ has shown promise in treating MDR-TB, its use is constrained by significant drawbacks, such as potential cardiac toxicity, QTc prolongation, phospholipidosis, hepatotoxicity, and high costs (44). These challenges underscore the critical need for alternative treatments that are safer and more affordable. WX-081 is a novel drug against DR-TB currently in Phase III clinical trials. Compared to BDQ, WX-081 retains similar anti-tuberculosis activity while demonstrating a superior safety profile, positioning it as a promising replacement for BDQ. However, despite its potential, the mechanism of action of WX-081 has not yet been systematically studied. This lack of detailed understanding highlights the need for further research to elucidate how WX-081 exerts its therapeutic effects, which could optimize its use and contribute to more effective DR-TB treatment strategies in the future. In this study, we discovered that WX-081 exerts its antibacterial effects by targeting the c subunit of ATP synthase, *atpE*, and disrupting the PMF, leading to the collapse of the bacterial energy metabolism.

To determine the molecular mechanisms of WX-081, resistant strains are generated, the isolates obtained with G187C and C188T mutations in *atpE*, which have also been previously reported in clinical BDQ-resistant strains (45), and *atpE* mutations are associated with high-level resistance (WX-081 MIC of >1 µg/mL) compared to *Rv0678* mutations. Our findings indicate that *Rv0678* mutations might represent an initial, transient phase in the development of low-level resistance, potentially progressing to high-level resistance consequent to stable *atpE* mutations (46). Furthermore, WX-081 also displays protonophore activity. As shown above, the uncoupling effect of Mtb by WX-081 is electroneutral, collapsing the pH gradient but not the membrane potential. This further substantiates the notion that diarylquinoline compounds appear to transport ions across the lipid bilayer in mycobacterial inner membrane vesicles to uncouple the PMF (35).

Host immune modulation enhances pathogen killing while attenuating pathological damage to the host, which is important for combating Mtb infections, particularly against drug-resistant Mtb (47). Here, we investigated the effect of WX-081 on host innate immunity and found that WX-081 promotes the expression of cytokines and type I interferon in macrophages stimulated with LPS, H37Rv, and WX-081r-Mtb, such as TNF-α, IL-2, IL-12, and IFNγ, a similar effect to that of BDQ. Previous studies have reported that BDQ enhances autophagy by upregulating the autophagy-related transcription factor, TFEB (48). In this study, we also observed that WX-081 induces the downregulation of the mTOR signaling pathway, which is typically an inhibitor of autophagy. Additionally, we discovered that BDQ significantly upregulates and activates the host’s innate immune response, enhancing the expression of cytokines. This finding has not been reported before and represents a novel discovery in our research. The immune-activating properties of WX-081 significantly enhance its intracellular antibacterial activity. Furthermore, treatment with WX-081 also suppresses the growth of drug-resistant strains through its immunomodulatory effects. This dual mechanism—directly targeting the bacteria while simultaneously leveraging the host immune system—underscores the potential of WX-081 as a powerful therapeutic agent against both drug-sensitive and drug-resistant tuberculosis.

## MATERIALS AND METHODS

### Chemicals

Clofazimine (CFZ), rifampicin (RFP), and isoniazid (INH) were purchased from Sigma-Aldrich (Missouri, USA). Bedaquiline was purchased from Biochempartner (Shanghai, China). Sudapyridine (WX-081) was provided by Shanghai Jiatan Pharmaceutical Technology. BAY11-7082 (M2040), SP600125 (M2076), and U0126 (M1977) were purchased from AbMole (Guangzhou, China).

### Bacterial strains and culture medium

*Mycobacterium tuberculosis* H37Rv (ATCC 27294) and BDQ-resistant strains (*atpE*^D28G^) for this study were obtained from the National Clinical Laboratory on Tuberculosis in Beijing Chest Hospital (49). All strains were grown in Middlebrook 7H9 broth (BD, USA) supplemented with 10% (vol/vol) oleic acid-albumin-dextrose-catalase (OADC) (Becton, Dickinson, USA), 0.2% (vol/vol) glycerol, and 0.05% Tween 80.

### Cell lines and plasmids

HEK293T (ATCC CRL-3216, RRID: CVCL_0063) and J774A.1 (ATCC TIB-67, RRID: CVCL_0358) were obtained from the American Type Culture Collection (ATCC). HEK293T cells and J774A.1 cells were maintained in Dulbecco’s modified Eagle’s medium (Gibco, USA) supplemented with 10% fetal bovine serum (FBS) (Gibco, USA). All cells were incubated at 37°C in a humidified 5% CO_2_ atmosphere.

Luciferase reporter assay plasmids for pRL-TK, Gal4-Elk, Gal4-Luc, pFA-cJun, RacL61, pNF-κB-luc, and RasV12 were kindly provided by Cuihua Liu (Institute of Microbiology, Chinese Academy of Sciences).

### Minimum inhibitory concentration

The MABA method was used to determine the MICs of WX-081 and BDQ against the *M. tuberculosis* H37Rv strain and drug-resistant isolates. According to the MABA (50), serial twofold dilutions of the test compounds were added to each well of a 96-well microplate, followed by the bacterial suspension containing 2 × 10^5^ CFU. The plate was incubated at 37°C in 5% CO_2_ for 7 days. A mixture of alamarBlue (Bio-Rad) was added to each culture well, after incubation at 37°C for a further 24 h. A change from blue to pink or purple indicated bacterial growth (51). The MIC value was determined as the lowest drug concentration of antibiotic that prevented a color change from blue to pink.

### Isolation of spontaneous mutants

H37Rv was cultured in Middlebrook 7H9 broth with glycerol and OADC supplementation at an initial density of 1  ×  10^4^ CFU/mL, and cultures were then incubated with shaking for a further 3 weeks to log phase (52). A total of 10^8^ CFU bacteria were plated on selective agar containing the test compound at 4× MIC or 8× MIC. Four weeks after inoculation, resistant strains growing on drug-containing plates were harvested, and the MICs for all strains were determined.

### Mutant selection under different concentrations of WX-081

Cultures containing approximately 10^8^ CFU/mL were exposed to WX-081 at 0.125 µg/mL (1× MIC) in solid agar plates for 3–4 weeks at 37°C. Clones grown on the drug-containing plates were transferred to a 7H10 medium containing 0.3 µg/mL (2× MIC) drug. Using this method, the highly resistant strains capable of survival were selected, and isolation clones were then grown in 7H9 broth for MIC determination as described above.

### PCR and DNA sequencing

Genomic DNA of the resistant mutants to WX-081 was isolated as previously described (53). PCR amplification of genes of all the mutants was performed using the following primers: *atpE* forward primer (5′-CCATCAAGGAGGATAAGGAAA-3′) and reverse primer (5′-CGAAAGTGCCAATGACAGC-3′); *Rv067*8 forward primers (5′-CGGCTATTTCGAGTCCAGG-3′) and reverse primers (5′-GCAACCGCAT CAACAAGG-3′); *pepQ* forward primer (5′-ctgccacgcgttgatcaat-3′) and reverse primer (5′-cttgaagtcagcagtggtcg-3′). Genome sequencing analysis was performed by Beijing ReboXingke Biotechnology. The sequences of these genes of all clones were compared with those of the wild-type strain to identify mutations.

### Cellular ATP assay

Cultures of wild-type or mutant *M. tuberculosis* (about 10^6^ CFU/mL) were incubated for 24 h with different concentrations of WX-081, 0.03 µg/mL BDQ (1/2× MIC), or 100 μM DCCD. The mixture was lysed on ice for 10 min, and cells were then removed by centrifugation. Total ATP was determined using an ATP assay kit (Beyotime, China) based on a bioluminescence technique according to the instructions.

### Preparation of *M. smegmatis* inverted membrane vesicles

IMVs were prepared according to a previously described method. Briefly, about 5 g (wet weight) of *M. smegmati*s was resuspended in 20 mL of 50 mM 3-(N-morpholino)propanesulfonic acid (MOPS) buffer (pH 7.5) containing 2 mM MgCl_2_ and protease inhibitors (Beyotime, China). Lysozyme was added to a final concentration of 1.2 µg/mL, and the suspension was stirred for 45 min at room temperature and additionally supplemented with 300 µL 1 M MgCl_2_ and 50 µL DNase I (Thermo Fisher), then continued stirring for another 15 min at room temperature. All subsequent steps were performed at 4°C unless otherwise stated. The cells were broken by three passages through a pre-cooled French pressure cell at 20,000 psi (Thermo Electron, 40K). The lysate was centrifuged at 5,000 × *g* for 20 min to remove unbroken cells. The cell-free supernatant was centrifuged at 45,000 × *g* for 1 h to harvest membrane vesicles. The reversed membrane fraction is resuspended in an appropriate volume of 50 mM MOPS buffer (pH 7.5) containing 2 mM MgCl_2_.

### ATP synthesis assay

The ATP synthesis activity was determined as previously described (54). Briefly, IMVs were diluted to a concentration of 50 µg protein/mL with 50 mM MOPS (pH 7.5 with 10 mM MgCl_2_). Membrane vesicles were pre-incubated with WX-081 or control inhibitors under stirring conditions at room temperature for 10 min. Subsequently, 2.5 mM NADH was added and further incubated with vigorous shaking for 1 min. The reaction was started by adding 1 mM ADP and 10 mM potassium phosphate. A volume of 50 µL of this mixture was added to a 96-well plate. After the addition of 50 µL luciferase reagent, the ATP level was measured as described above.

### Determination of ΔpH collapse with IMVs

Collapse of ΔpH was determined in *M. smegmatis* IMVs as previously described (36). The pH-sensitive, fluorescent dye ACMA was purchased from Aladdin (Shanghai, China). IMVs were diluted to 0.1125 mg/mL in 10 mM HEPES (pH 7.5), 100 mM KCl, 5 mM MgCl_2_ and added to a black-wall, 96-well plate (Corning). IMVs were pre-incubated with 2 µM ACMA at 37°C for 30 min, and the baseline 410 nm excitation/460 nm emission fluorescence was measured using a Biotek Synergy HT multi-mode plate reader. IMVs were energized with 5 mM NADH and incubated until ACMA fluorescence was quenched due to the generation of ΔpH. Thereafter, IMVs were treated with DMSO, 15 µM WX-081, 15 µM BDQ, and 15 µM CCCP and monitored to test for fluorescence reversal if ΔpH collapsed.

### Determination of Δψ collapse by DisC_3_(5)

Bacterial cells were collected and resuspended with PBS (pH 7.4, plus 5 mM glucose) to obtain an optical density at 600 nm (OD600) of 0.5. Membrane potential sensitive probe 3,3′-dipropylthiodicarbocyanine iodide [DiSC_3_(5), Aladdin, Shanghai, China] was diluted to 0.5 µM and then added to the culture. After 30 min, once fluorescence was quenched, the bacteria were treated with DMSO, 15 µM WX-081, 1 µM WX-081, 15 µM BDQ, or 1 µM BDQ, while 1% SDS was used as a positive control. Fluorescence intensity was monitored for 60 min at an excitation wavelength of 622 nm and an emission wavelength of 670 nm in the presence of both drugs.

### Protein ligand docking

Molecular docking studies were carried out to determine the interaction between the *atpE* and the selected ligands using the AutoDock 4.2 tool. The crystal structure of ATP synthase was obtained from the RCSB database using source code 4V1F. The receptor protein was kept rigid for docking, while the pharmacological component consisted of a flexible molecule. The docking grid documents were generated by AutoGrid of sitemap, and AutoDock Vina (1.2.0) was used for docking simulation (55). To represent the interaction between the ligand and the protein, BIOVIA Discovery Studio software was used to select the position with the lowest binding energy (56). Finally, the root mean square deviation (RMSD) was calculated, and the protein-ligand complex was analyzed using Pymol.

### Dual luciferase reporter assays

Activation of the NF-κB, AP-1, and ELK promoters was detected using the luciferase reporting assay kit (Promega, USA). HEK293T cells were seeded into 24-well plates and incubated for 12 h before being transfected with the appropriate plasmids. To assess NF-κB activity, pNF-κB-Luc (0.5 µg) and pRL-TK (25 ng) were transfected for 6 h, then the medium was replaced with Dulbecco’ s modified Eagle’ s medium (DMEM) containing 10% FBS, along with either drugs or DMSO. After a further 18 h, 20 ng/mL TNF-α (Sigma) was added for a further 6 h. For analysis of AP-1 activity, cells were transfected with Gal4-luc (0.45 µg), pFA-cJun (0.15 µg), RacL61 (0.25 µg), and pRL-TK (25 ng) for 6 h, after which the medium was replaced with a solution containing 5 µg/mL of drugs or DMSO. For the detection of Elk, the cells were transfected with Gal4-luc (0.3 µg), Gal4-Elk (0.3 µg), pRL-TK (25 ng), and RasV12 (5 ng) as described above.

### Quantitative RT-PCR analysis

Total RNA was extracted from cells using TRIzol (Invitrogen, USA) and reverse-transcribed into cDNA, which was then analyzed by quantitative RT-PCR (RT-qPCR) with SYBR Green Real-time PCR Master Mix (Yeasen, China) with specific primers. The primers used were as follows: IL-6-forward: TACCACTCCCAACAGACC reverse: CATTTCCACGATTTCCCAGA; IL-1β-forward: GCCACCTTTTGACAGTG ATG reverse: TGATGTGCTGCTGCGAGA; TNF-α-forward: TCTCATTCCTGCTT GTGG reverse: ACTTGGTGGTTTGCTACGA; GAPDH-forward: CAAATTCAA CGGCACAGTCA reverse: TTAGTGGGGTCTCGCTCC; IFNα-forward: CCTGTGT GATGCAGGAACC reverse: TCACCTCCCAGGCACAGA; IFNβ-forward: ACTAG AGGAAAAGCAAGAGGA reverse: CTGGTAAGTCTTCGAATGATG; IL-12-forward: CTGTGCCTTGGTAGCATCTATG reverse: GCAGAGTCTCGCCATT ATGA TTC.

### Infection of macrophages

J774A.1 cells were seeded at 5 × 10^5^ cells/well in a complete medium (10% FBS) in a 24-well plate. Cells were washed three times and infected with WX-081r-Mtb at an MOI of 5 for 4 h at 37°C with 5% CO_2_. After 4 h, infected cells were washed thoroughly with fresh medium to remove extracellular bacteria, and the medium was replaced with 0.5 µg/mL or 1 µg/mL WX-081 for a further 48 h, a volume of DMSO or BDQ as control. Macrophages were lysed using SDS lysis buffer (Sigma-Aldrich). The number of viable bacteria in lysates was determined by plating 10-fold serial dilutions on MB-7H10 agar at 37°C for 3–4 weeks.

### Statistical analysis

All data were presented as the mean ± standard deviation. Statistical analysis was performed using GraphPad Prism 8 software (GraphPad Software, Inc.). The experimental group means were compared to the untreated group by two-sided *t*-test. *P* value of 0.05 was considered significant.

## Data Availability

The data presented in this study are available from the corresponding author upon reasonable request.
